# Mechanical Properties and Thermal Conductivity of Ytterbium-Silicate-Mullite Composites

**DOI:** 10.3390/ma13030671

**Published:** 2020-02-03

**Authors:** Jie Xiao, Wenbo Chen, Liangliang Wei, Wenting He, Hongbo Guo

**Affiliations:** 1 School of Materials Science and Engineering, Beihang University, No. 37, Xueyuan Road, Beijing 100191, China; xiaojie@buaa.edu.cn (J.X.); justinchen123@163.com (W.C.); weill@buaa.edu.cn (L.W.); 2 Key Laboratory of High-temperature Structural Materials & Coatings Technology (Ministry of Industry and Information Technology), Beihang University, No. 37, Xueyuan Road, Beijing 100191, China

**Keywords:** mullite, Yb_2_SiO_5_, Yb_2_Si_2_O_7_, Vickers hardness, fracture toughness, thermal conductivity

## Abstract

Various Ytterbium-Silicate-Mullite composites were successfully fabricated by adding Yb_2_SiO_5_ into mullite ceramics and then using pressureless sintering at 1550 °C. The influence of Yb_2_SiO_5_ addition on the microstructure, mechanical properties, and thermal conductivity of ytterbium-silicate-mullite composites was investigated. Results show that the composites mainly consisted of a mullite matrix and some in situ formed Yb_2_Si_2_O_7_ and Al_2_O_3_ phases. By the addition of Yb_2_SiO_5_, the Vickers hardness of composites reached ~9.0 at an additive concentration of 5 mol %. Fracture toughness increased to ~2.7 MPa·m^1/2^ at the additive concentration of 15 mol %, owing to the trans-granular fracture and crack deflection of the pinning effect of the Al_2_O_3_ phase in the composites. With the increase of the Al_2_O_3_ phase in the composite, the thermal conductivity for the 15YbAM reached around 4.0 W/(m·K) at 1200 °C. Although the thermal conductivity increased, it is still acceptable for such composites to be used as environmental barrier coatings.

## 1. Introduction

In order to achieve higher efficiency and stronger thrust of aero engines, demand for higher turbine inlet temperatures is increasing. Technology improvements in cooling, structural materials, and coatings are required to allow higher inlet temperatures [1]. As the temperature capability of the latest Ni-based super-alloys approaches their intrinsic limit, it is rather difficult to significantly improve the temperature capability. Therefore, during the last decades, ceramic matrix composites (CMCs) that can endure even higher service temperatures have become the most promising candidates for the hot components of next generation aero engines, especially SiC/SiC CMCs.

A major drawback of SiC/SiC CMCs is the lack of environmental durability in combustion environments. The reaction of water vapor with silica scale grown on the composite would produce volatile silicon hydroxide, which tends to cause the rapid recession of SiC/SiC in high-pressure and high-velocity combustion environments [2,3,4]. Thus, environmental barrier coatings (EBCs) were developed to prevent environmental corrosion of SiC/SiC. To meet the key requirements of thermal expansion, water vapor stability, chemical compatibility, phase stability, and adherence, multi-layer coating systems have been designed and investigated. Currently, a typical EBC is usually a tri-layer system, consisting of a rare earth silicate (Yb, Sc, Er, etc.) top layer, a mullite (3Al_2_O_3_·2SiO_2_) intermediate layer and a Si bond layer [5,6,7,8,9,10,11]. However, due to the mismatch of the coefficient of thermal expansion (CTE) between each layer, the formation and propagation of cracks become the bottleneck in the service of tri-layer EBCs [12,13,14]. Once cracks propagate through layers, the hot gas can infiltrate into the underlying layers through the cracks. As the result, the exposed Si bond coat will be preferentially oxidized to form β-cristobalite (thermally grown oxide, TGO) [15,16]. Upon thermal cycling, cristobalite transforms from a β to α phase, accompanied by a large volume shrinkage (about 4.5%, as reported [17]), which could cause severe micro-fractures and eventually result in spallation of the coatings. Thus, restraining the crack propagation plays an important role in prolonging the lifetime of EBCs.

As we know, the thermal shock resistance of the mullite layer is relatively poor, partially due to its low fracture toughness, which limits its application. Many efforts have been made to improve the fracture toughness, including the reinforcement of mullite composites with Al_2_O_3_, ZrO_2_, and SiC particles [18,19,20], carbon fibers [21], and mullite whiskers [22]. On top of the mullite, ytterbium monosilicate (Yb_2_SiO_5_, YbMS) has been extensively used as the top layer in EBCs due to its chemical stability in steam, low CTE, and phase stability at high temperature [23,24,25,26]. According to the Al_2_O_3_-Yb_2_O_3_-SiO_2_ phase diagram [27], the Al_2_O_3_ phase can be formed in situ around the mullite phase due to the interaction between Yb_2_SiO_5_ and mullite at 1550 °C. Therefore, using a candidate of coating material as an additive for mullite to improve its fracture toughness has attracted our interest.

Following the above idea, in this work, a series of mullite composites with different amounts of Yb_2_SiO_5_ (0~15 mol %) were prepared. The effects of Yb_2_SiO_5_ addition on the mechanical properties and thermal conductivity of the composites were investigated, and associated mechanisms are discussed.

## 2. Experimental and Characterization Methods 

### 2.1. Specimen Preparation

A series of mullite composites with different concentrations of Yb_2_SiO_5_ (0, 5, 10, and 15 mol %), marked as mullite, 5YbAM, 10YbAM, and 15YbAM, respectively, were fabricated by a solid-state method. The starting powders, mullite and Yb_2_SiO_5_, were synthesized by the chemical co-precipitation method. In the preparation, the mixed powders were ball milled in ethanol for 10 h, then dried at 120 °C for 24 h. After cold pressing (500 MPa, 3 min), and sintering at 1550 °C for 4 h in an air furnace for densification, grinding and polishing were performed to obtain a specimen with a diameter of 10 mm and a thickness of 2 mm.

### 2.2. Structure and Composition

The bulk densities of the sintered samples were determined by Archimedes’ method. Phase constituents were identified by X-ray diffraction (XRD, D/Max 2200PC, Cu/Kα, Rigaku, Tokyo, Japan) at a scanning 2θ speed of 6°/min. For Rietveld refinement analyses, XRD data were collected in a D/Max 2500PC diffractometer (Rigaku, Tokyo, Japan) using Cu Kα radiation at a scanning 2θ speed of 0.02°/s in step increment mode. Before microstructure observation, the samples were grinded, finely polished and hot etched for 2 h at 1450 °C. Microstructures were characterized by a scanning electron microscopy (SEM, Gemini SEM 300, Zeiss, Oberkochen, Germany) equipped with an energy dispersive spectrometer (EDS).

### 2.3. Mechanical Properties 

The elastic modulus was measured by the ultrasonic method (Ultrasonic Pulser/Receiver Model 5058 PR, Olympus, Waltham, MA, USA). Young’s modulus (*E*) and Poisson’s ratio (*υ*) can be calculated by the following equations [28,29]:

(1) $$E=\rho V_{t}^{2}\frac{3V_{l}^{2}-4V_{t}^{2}}{V_{l}^{2}-V_{t}^{2}}$$

(2) $$\upsilon =\frac{V_{l}^{2}-2V_{t}^{2}}{2V_{l}^{2}-2V_{t}^{2}}$$

where *ρ*, *V_t_*, and *V_l_* are density, transverse and longitudinal acoustic velocities, respectively. Since the ceramic samples were not fully dense, to eliminate the effect of pores on Young’s modulus, the elastic modulus of a fully dense specimen (*E*_0_) can be modified with porosity (ϕ) by the following equation [30]:

(3) $$E_{0}=\frac{E}{{\left(1-\phi^{2/3}\right)}^{1.21}\;}$$

Vickers hardness (*Hv*) and fracture toughness (*K_IC_*) were measured by a micro-hardness tester with an applied load (*p* = 196 N) for 15 s at room temperature. *Hv* and *K_IC_* and the critical energy release rate (*G_IC_*) can be assessed from the following equations [31]:

(4) $$H_{v}=0.464\frac{P}{d^{2}}\;$$

(5) $$K_{IC}=0.016\sqrt{\frac{E}{H_{v}}}\cdot \frac{P}{c^{1.5}}$$

(6) $$G_{IC}=K_{IC}^{2}\frac{1-\nu^{2}}{E}$$

where *d*, *c*, and *υ* are the half length of the indent diagonal, the crack length, and the Poisson’s ratio, respectively. Ten indents were performed on each specimen.

### 2.4. Thermal Conductivities

The thermal diffusivity (*α*) was measured by a laser flash analyzer (Netzsch LFA 427, Germany) from 25 to 1200 °C in an Ar atmosphere. The heat capacities (*C*) of the mullite (3Al_2_O_3_·2SiO_2_) and Yb_2_SiO_5_ were calculated from the total heat capacities of their constituent oxides (Al_2_O_3_, SiO_2_, and Yb_2_O_3_) according to the Neumann-Kopp law [32]. Thermal conductivity (*k’*) can be calculated by the following equation [33]:

(7) $$k^{\prime }=\alpha \cdot \rho \cdot C$$

To eliminate the influence of the porosity, the thermal conductivities of fully dense specimens (*k*) were calibrated by [30,34]:

(8) k′/k=1−4/3ϕ 

## 3. Results and Discussion

### 3.1. Microstructure Characterization

**Figure 1:**
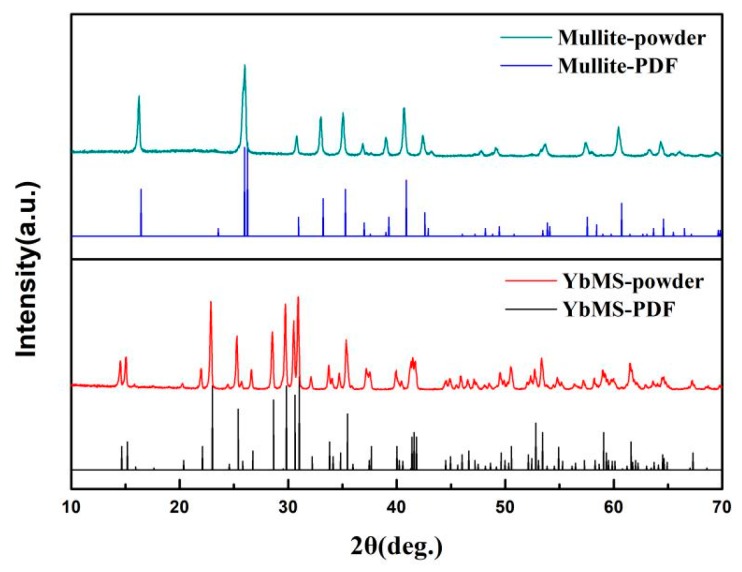
XRD patterns of the starting powders.

**Figure 2:**
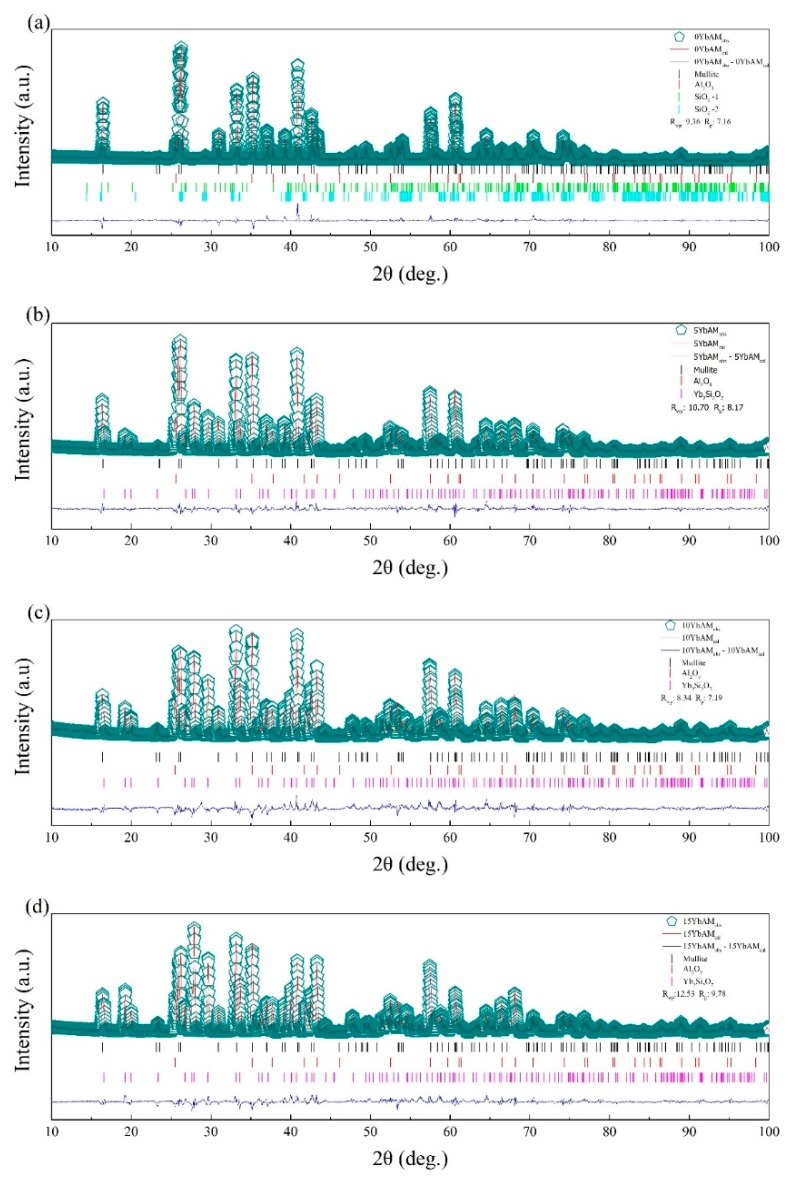
Refined XRD patterns of (**a**) mullite, (**b**) 5YbAM, (**c**) 10YbAM, and (**d**) 15YbAM.

Figure 1 shows the XRD patterns of the starting powders. Peaks of mullite and Yb_2_SiO_5_ match the standard powder diffraction files (PDF) perfectly without additional peaks, which means the synthesized mullite and Yb_2_SiO_5_ possess high phase purity. Figure 2 presents the refined XRD patterns of mullite and YbAM specimens. The mullite sample is basically composed of a mullite phase. Only a small amount of Al_2_O_3_ phase is detected, which might precipitate from the mullite, as reported by others [35,36,37]. All the YbAM samples consisted of mullite, Yb_2_Si_2_O_7_, and Al_2_O_3_. With increasing concentrations of Yb_2_SiO_5_ in the starting materials, the amount of Yb_2_Si_2_O_7_ and Al_2_O_3_ in the YbAM samples increased while the amount of mullite decreased. One possible reason is that the mullite matrix partially decomposed into Al_2_O_3_ and SiO_2_ during the sintering process, and then SiO_2_ reacted with Yb_2_SiO_5_ to form Yb_2_Si_2_O_7_. It may also be that Yb_2_SiO_5_ reacted with SiO_2_ directly from mullite, thus leading to the precipitation of Al_2_O_3_ from mullite. Table 1 lists the phase contents of all the samples. The formation of Al_2_O_3_ can be described by the following equation:

(9) $$2{\mathrm{Yb}}_{2}{\mathrm{SiO}}_{5}+3{\mathrm{Al}}_{2}O_{3}\cdot 2{\mathrm{SiO}}_{2}\to 2{\mathrm{Yb}}_{2}{\mathrm{Si}}_{2}O_{7}+3{\mathrm{Al}}_{2}O_{3}$$

**Figure 3:**
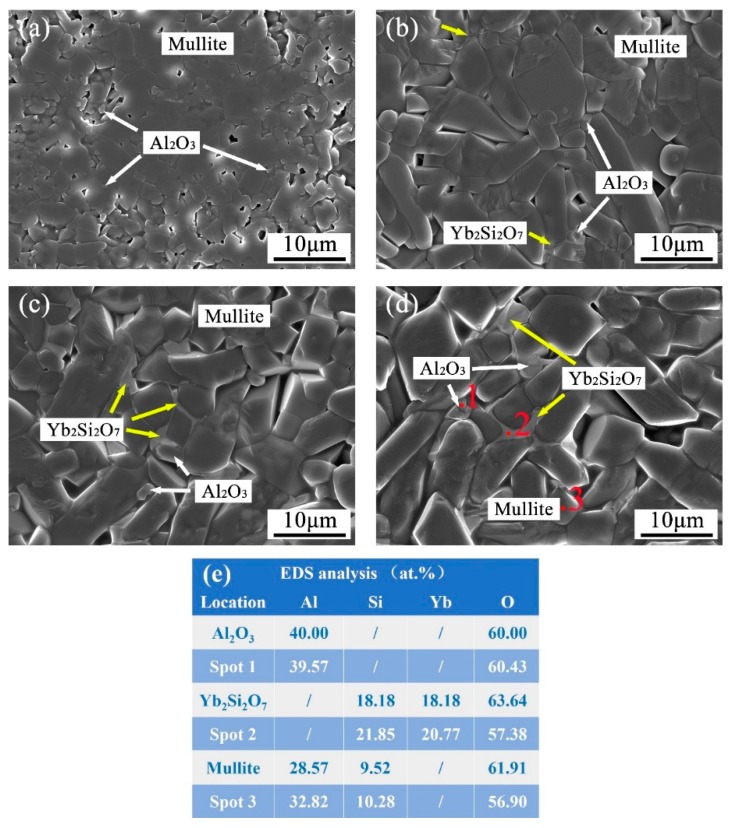
SEM micrographs of hot corrosion surface of (**a**) mullite, (**b**) 5YbAM, (**c**) 10YbAM, (**d**) 15YbAM samples and (**e**) energy dispersive spectrometer (EDS) analysis of three grains in (**d**).

The microstructure of the above samples and the composition of different phases determined by EDS analyses are shown in Figure 3. Combined with the results of XRD analyses, the phases were determined and marked in Figure 3. The grain size of the mullite phase in the mullite sample was 2–5 μm (Figure 3a). In contrast to the mullite sample, the grains of mullite in the YbAM samples were apparently coarser, with a size of 5–15 μm (Figure 3b–d). In addition, some light grey phases were observed around the mullite grains, which were determined to be an Yb_2_Si_2_O_7_ phase that is much finer than the mullite phase. This might be attributed to the formation of a liquid phase during sintering at 1550 °C, according to the Al_2_O_3_-Yb_2_O_3_-SiO_2_ phase diagram [27]. Furthermore, it can be confirmed from the shape of the Yb_2_Si_2_O_7_ phase that the liquid phase was generated during sintering, and thus the flowable liquid filled the interspace between the mullite grains after cooling down. In addition, some fine Al_2_O_3_ phases of several micrometers are located between the coarser mullite phases. This flowable characteristic of the Yb_2_Si_2_O_7_ phase at high temperatures makes the YbAM composites a promising self-healing material when used as environmental barrier coating (EBC).

### 3.2. Mechanical Properties

Table 2 lists the density *ρ*, acoustic velocities *V*, and mechanical properties of the YbAM composite samples. Compared to that of the mullite sample, the density of the YbAM samples is relatively higher, mainly due to the formation of a liquid phase at high temperatures accelerating the densification. The Young’s modulus (*E*_0_) of the mullite sample is about 224 GPa, which is similar to the reported result in Reference [38]. With increasing the content of Yb_2_SiO_5_ from 5 to 15 mol %, the Young’s modulus became higher, in the range of 249–265 GPa. This implies the YbAM samples have an enhanced resistance to tensile stress. The Poisson’s ratios (υ) of the YbAM samples range from 0.24–0.27, similar to most ceramic samples.

**Figure 4:**
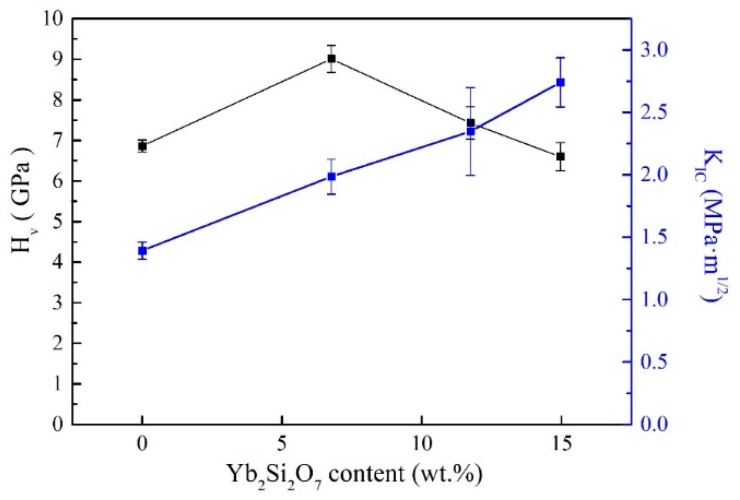
Vickers hardness and fracture toughness of the samples.

The Vickers hardness and fracture toughness of the mullite and YbAM composite samples are illustrated in Figure 4. The hardness of the samples increased to 9 GPa with the increase in concentration of Yb_2_SiO_5_ to 7 wt %, however, this decreased to 6.5 GPa as the concentration of Yb_2_SiO_5_ was increased to 15 wt %. In general, the hardness of ceramics is highly influenced by the phase constituents, the grain size and the porosity of the materials. The newly formed Al_2_O_3_ phase in the YbAM composite shows much higher Vickers hardness (*Hv*: 18–21 GPa [39,40]) than that of the mullite phase (*Hv*: 6.9 ± 0.2 GPa). In the current case, the fine Al_2_O_3_ grains are distributed between the grain boundaries and can hinder the local deformation, thus improving the hardness. Therefore, the Vickers hardness of the 5YbAM sample increased to 9.0 ± 0.3 GPa, around 30% higher than that of the mullite sample. However, on the other hand, an Yb_2_Si_2_O_7_ phase was also generated in the YbAM composites, the hardness of which is relatively lower (*Hv*: 6–7 GPa [26]). As the result, the Vickers hardness of the composite reduced gradually when the content of the Yb_2_Si_2_O_7_ phase increased from 5YbAM to 15YbAM.

**Figure 5:**
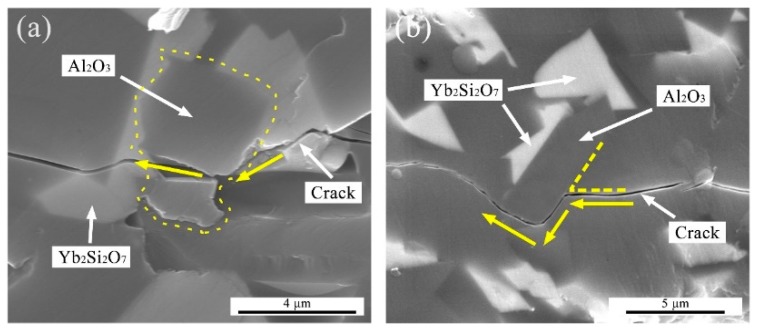
SEM micrographs of different crack penetration modes: (**a**) trans-granular fracture and (**b**) crack deflection.

The fracture toughness of the mullite sample was 1.4 ± 0.1 MPa·m^1/2^. After adding Yb_2_SiO_5_, the fracture toughness of the composites nearly doubled to 2.7 ± 0.2 MPa·m^1/2^ for the 15YbAM. Crack propagation paths induced by indentation in the 15YbAM sample were observed by SEM as shown in Figure 5. It can be clearly seen that the fine Al_2_O_3_ and Yb_2_Si_2_O_7_ grains were distributed in the composite, working as pins in the microstructure. Since the elastic modulus of Al_2_O_3_ (about 380 GPa [41,42]) is much higher than that of mullite (about 224 GPa, Table 2), the cracks that penetrated Al_2_O_3_ grains (Figure 5a) consumed much more energy than the cracks that did not penetrate Al_2_O_3_ grains. Meanwhile, the cracks which propagated along Al_2_O_3_ grain boundaries also consumed more energy than that consumed by the cracks that penetrated through mullite grains (Figure 5b). According to Griffith’s energy release theory, the critical energy release rate (*G_IC_*) is used to quantify the energy released by crack propagation, in other words, the required energy for the cracks’ propagation. As given in Table 2, the *G_IC_* increased nearly by 270% from the mullite (~10 J∙m^−2^) to the 15YbAM (~38 J∙m^−2^). These results strongly indicate that the Al_2_O_3_ formed in situ in the YbAM composites is highly effective in toughening mullite ceramics. This improved fracture toughness could prolong the thermal cycling lifetime of YbAM composites, which is very meaningful for its application as an EBC material.

### 3.3. Thermal Conductivity

**Figure 6:**
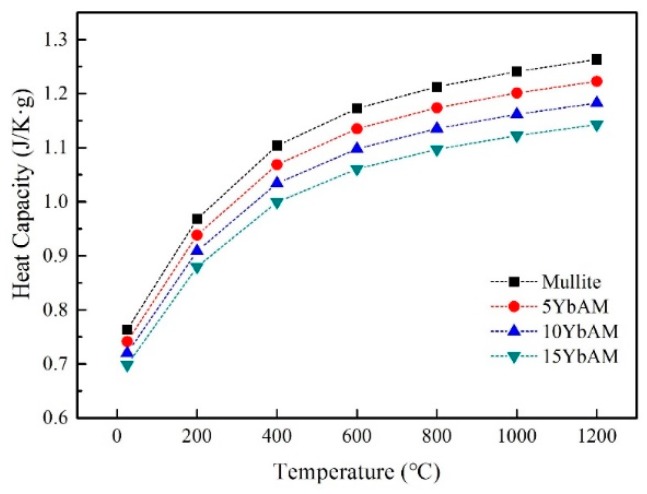
The heat capacity of the samples at 25 to 1200 °C.

**Figure 7:**
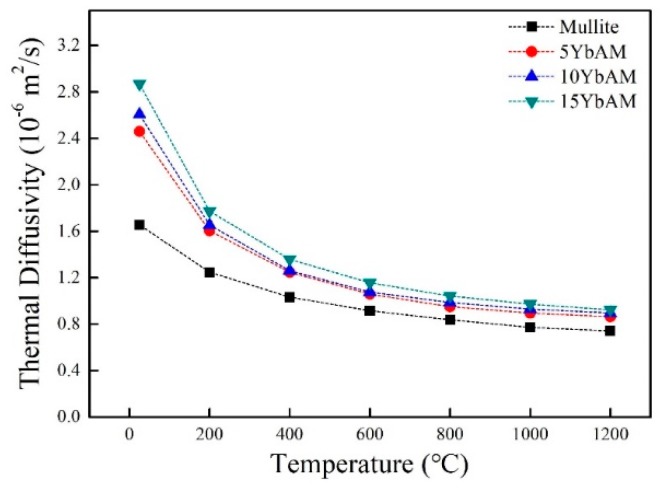
Thermal diffusivity of samples as a function of temperature.

The thermal conductivity of ceramic is an important property for its application as a thermal protection material. Here, thermal conductivities of the samples were calculated using Equations (7) and (8) in the temperature range from 25 to 1200 °C. The heat capacity (*C_p_*) of the samples was calculated and plotted in Figure 6. As one can see, the heat capacities of all samples dramatically increased along with increasing temperature. The thermal diffusion (*α*) was measured based on the Cape–Lehman model [43], which includes both phonon scattering and photon radiation contributions to evaluate the total thermal diffusivity. The thermal diffusivity of the samples as a function of temperature is plotted in Figure 7. With the temperature rising, the thermal diffusivity of the mullite sample decreased from 1.7 × 10^−6^ m^2^/s at 25 °C to 0.7 × 10^−6^ m^2^/s at 1200 °C, similar to other reports [44,45]. Such declining trends can be also observed in other YbAM composites. Apparently, at the same temperature, there is a positive linear relationship between the thermal diffusivity and the concentration of Yb_2_Si_2_O_7_, which indicates that the additive plays an important role in the thermal diffusivity of the YbAM composites.

**Figure 8:**
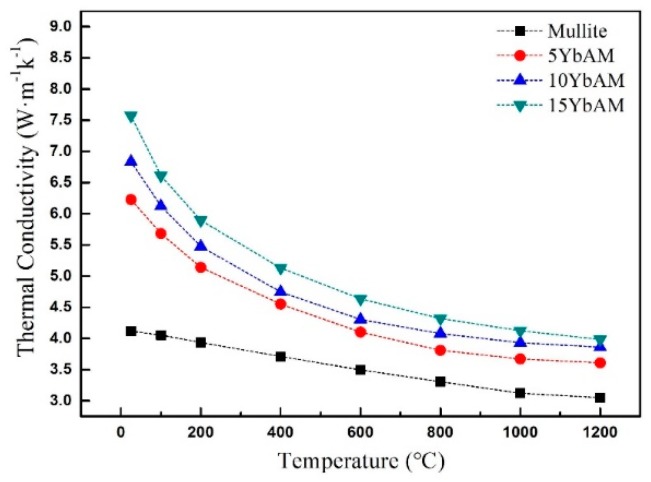
Thermal conductivity of samples as a function of temperature.

The thermal conductivity (*k*) of the samples is calculated by Equations (7) and (8), based on the above calculated *C_p_* and measured *α*. Obviously, the thermal conductivities decrease fast at low temperatures (RT to 400 °C) and then decrease more gradually as the temperature rises, as shown in Figure 8. From 25 °C to 1200 °C, the *k* of the mullite sample decreased from 4.1 W/(m·K) to 3.0 W/(m·K). Likewise, the *k* of the 15YbAM sample decreased by 47% from 7.6 W/(m·K) at 25 °C to 4.0 W/(m·K) at 1200 °C. Such a reduction of *k* was mainly due to scattering of phonons. According to the Debye phonon gas theory, thermal conduction in insulation material can be considered as the transport of phonons. Due to the fact that the real crystal is not homogeneous and perfect, the interaction of phonons with various defects (oxygen vacancies, crystal cell structure, etc.) causes the scattering of phonons in the propagation. Moreover, non-harmonic lattice vibration and interaction among phonons can also scatter phonons. These scatterings reduce the mean free path of phonons, and thus result in the decrease of thermal conductivity, according to the following equation:

(10) $$k_{p}=\frac{1}{3}C_{V}\cdot V\cdot l\;$$

where *k_p_* is the phonon thermal conduction, *C_V_* the specific heat capacity per unit of volume, *V* the average sound velocity, and *l* the mean phonon free path.

It can be also seen from Figure 8 that the samples with higher concentrations of Yb_2_Si_2_O_7_ reveal increased thermal conductivities. For instance, the thermal conductivity at 1200 °C increased from 3.6 W/(m·K) for the 5YbAM sample to 4.0 W/(m·K) for the 15YbAM sample. The increase in the thermal conductivity could be due to the increase in the content of Al_2_O_3_ phase, which was formed in situ in the composite. The thermal conductivity of Al_2_O_3_ is ~10 W/(m·K) at 1200 °C [46], much higher than those of Yb_2_Si_2_O_7_ (~2 W/(m·K) [26]) and mullite.

## 4. Conclusions

The influence of the Yb_2_SiO_5_ addition on the microstructure, mechanical properties, and thermo-physical properties of the mullite-based composites were investigated. The main conclusions can be drawn as follows:

- With the addition of Yb_2_SiO_5_ to the mullite, Yb_2_Si_2_O_7_ and Al_2_O_3_ phases were formed in situ in the composites and distributed uniformly between the interspaces of mullite grains during the sintering of the composites at 1550 °C.
- Compared to the mullite sample, the Vickers hardness of the 5YbAM sample was increased by 30% to 9.0 ± 0.3 GPa. In particular, the fracture toughness of the 15YbAM was doubled to 2.7 ± 0.2 MPa·m^1/2^ due to the Al_2_O_3_ phase formed in situ.
- The samples with a higher content of Al_2_O_3_ phase revealed increased thermal conductivities. The thermal conductivity at 1200 °C increased from 3.6 W/(m·K) for the 5YbAM sample to 4.0 W/(m·K) for the 15YbAM sample.

In summary, the addition of Yb_2_SiO_5_ into mullite can induce the in situ formation of Al_2_O_3_ in composites, which leads to enhanced fracture toughness and moderately raises the thermal conductivity. Such a modification of mechanical properties and thermal conductivities of the mullite-based composite is promising for future application as EBCs.

The work on preparing this material as an environmental barrier coating is ongoing, and the effect of high temperatures on the microstructure and properties of the coatings will be studied.

## Figures and Tables

**Table 1:** Phase compositions and contents of the samples (wt %).

Samples	Mullite	Yb_2_Si_2_O_7_	Al_2_O_3_	SiO_2_
Mullite	81.61	-	13.88	4.51
5YbAM	72.33	6.77	20.91	-
10YbAM	60.49	11.75	27.76	-
15YbAM	52.29	14.96	34.74	-

**Table 2:** The density and mechanical properties of the samples.

Samples	ρ (g/cm^3^)	*V_t_* (m/s)	*V_l_* (m/s)	Porosity (%)	*E*_0_ (GPa)	υ	*G_IC_* (J∙m^−2^)
Mullite	2.91	4893	8505	8	224	0.25	~10
5YbAM	3.21	5149	8846	4.5	249	0.24	~18
10YbAM	3.24	5000	8625	8.3	261	0.25	~26
15YbAM	3.22	4781	8349	11.1	254	0.26	~38
